# Biological Active Ecuadorian Mango ‘Tommy Atkins’ Ingredients—An Opportunity to Reduce Agrowaste

**DOI:** 10.3390/nu10091138

**Published:** 2018-08-21

**Authors:** Jenny Ruales, Nieves Baenas, Diego A. Moreno, Carla M. Stinco, Antonio J. Meléndez-Martínez, Almudena García-Ruiz

**Affiliations:** 1 Department of Food Science and Biotechnology, Escuela Politécnica National, Quito 17-01-2759, Ecuador; jenny.ruales@epn.edu.ec; 2 Phytochemistry and Healthy Foods Lab., Department of Food Science and Technology, CEBAS-CSIC, Campus de Espinardo-Edificio 25, E-30100 Murcia, Spain; nbaenas@cebas.csic.es (N.B.); dmoreno@cebas.csic.es (D.A.M.); 3 Food Colour & Quality Lab., Department of Nutrition & Food Science, Universidad de Sevilla, Facultad de Farmacia, 41012 Sevilla, Spain; cstinco@us.es (C.M.S.); ajmelendez@us.es (A.J.M.-M.); 4 Laboratory of Epigenetics of Lipid Metabolism, Madrid Institute for Advanced Studies (IMDEA)-Food, CEI UAM + CSIC, 28049 Madrid, Spain

**Keywords:** mango by-products, lutein, β-carotene, α-tocopherol, mangiferin, food ingredients

## Abstract

Mango is a commercially important tropical fruit. During its processing, peel and seed kernel are discarded as waste but they could be recovered as an excellent and cost-effective source of health-promoting ingredients. This study aimed to characterize some of them, including carotenoids like the provitamin A β-carotene and lutein, with an interest beyond its role in eye health. Other health-promoting compounds like tocopherols and polyphenols were also evaluated, as well as the in vitro antioxidant capacity of mango by-products. Regarding isoprenoids, α-tocopherol was mainly found in the peels and carotenoids concentration was higher in the pulps. β-carotene was the most abundant carotene in pulp and seed kernel, whereas peel was the only source of lutein, with violaxanthin the most abundant xanthophyll in the different mango organs tested. With regard to polyphenols, peels exhibited greater variability in its phenolic composition, being the total content up to 85 and 10 times higher than the pulp and seed kernels, respectively. On the other hand, peels also stood out for being a very rich source of mangiferin. Seed kernels and peels showed higher antioxidant capacity values than the pulps. These results contribute to the valorization of mango by-products as new natural ingredients for the pharma and food industries.

## 1. Introduction

Mango (*Mangifera indica*) is considered one of the most consumed fresh fruits in the world, with extensive marketing and production taking place in 115 countries [1]. The global area of mangoes harvested is approximately 5.41 million hectares and its global production is 42.66 million metric tons (MMT) [2]. India, with a production of 18 MMT, is the world’s largest mango producer, whereas Mexico and the United States are the main mango exporter and importer, respectively [2]. There are several hundreds of cultivars of mango; but by its long shelf life, excellent ratings in handling and transport tolerance the cultivar Tommy Atkins is the most commercialized [1,3]. Ecuador, with a global area of mangoes harvested of 13,300 hectares and a production of 61,300 metric tons, is the second and sixth mango exporter to USA and to worldwide, respectively, being an important fruit in the Ecuadorian economy [2].

Apart of being consumed fresh, about 20% of mango are processed for products such as juices, desserts, mango jam, among others [4]. During processing, 33% of the fruit is removed in the form of waste, generating, as a result, several million tons per year of mango waste from factories [4,5]. The mango fractions discarded, peel and seed kernel (35–60% total weight of the fruit) are a source of pollution, among other reasons because they are prone to microbial spoilage causing objectionable odors and environmental problems [1,3]. However, the mango peel and seed kernel may be interesting because their high levels of health-enhancing substances, such as carotenoids, polyphenols, vitamins C and E and dietary fiber, among others [6]. The benefit effect of these phytochemicals may be associated with their antioxidant capacity, since in the pathogenesis of many chronic disease is involved the overproduction of oxidants [7]. On the other hand, various studies have described that efficient, inexpensive and environmentally friendly use of agri-food industry waste is highly cost-effective and minimizes environmental impact [8,9]. In this line, the characterization, recovery and utilization of valuable compounds from mango by-products is an important challenge, whose result would have a significant positive impact both at the environmental level (reduction of pollution of mango industry) and economically (contribution to more sustainable production in the food and pharmaceutical industries). In addition, the revaluation of mango peels and seed kernels as a natural bioactive ingredient for the industry, would have a positive socio-economical effect on Ecuadorian mango and tree fruit producing areas, contributing to a reduction of nutritional deficiencies and promoting health benefits, as well as reducing the environmental implications associated with the mango processing.

In this context, the main goal of the present work was to characterize and evaluate Ecuadorian mango by-products in their phytochemical composition: two types of isoprenoids, specifically carotenoids and α-tocopherol and polyphenols by RRLC (Rapid Resolución Liquid Chromatographic) and HPLC–DAD–ESI/MS^n^ (High-performance Liquid Chromatographic Diode-array detector Electrospray ionization Mass Spectometry), respectively and their total antioxidant capacity by ORAC (Oxygen Radical Absorbance Capacity) and DPPH (2,2,-diphenyl-2-picrylhydrazyl) methods, as well as by Folin–Ciocalteu assay. These evaluations were carried out to establish the potential applications of these mango discards or non-commercial products (peels and seed kernels) compared with the pulp (the main fraction consumed of the mango fruits) as a valuable source of natural ingredients and/or additives for the pharmaceutical and food industry.

## 2. Materials and Methods

### 2.1. Fruit Samples 

Mangoes (*Mangifera indica* L. cv. Tommy Atkins), were obtained in local markets in Quito (Ecuador). The fruits were selected free of damage. Two kilograms of samples were separated into peels, pulps and seed kernels and stored at −20 °C until freeze drying in an Alpha 2–4 LD drying manifold (Martin Christ Gefriertrocknungsanlagen GmbH, Osterode am Harz, Germany). Then, samples were ground as a fine powder and stored at −20 °C until analyses.

### 2.2. Standards, Chemicals and Solvents

The commercially available standards (+)-catechin and rutin (Quercetin-3-rutinoside) were acquired from Phytoplan GmbH (Heidelberg, Germany). Cyanidin 3-*o*-glucoside was purchased from Polyphenols (Sandnes, Norway). The β-carotene and β-cryptoxanthin were obtained from Sigma-Aldrich Chemie GmbH (Steinheim, Germany) and α-tocopherol was purchased from Calbiochem (Merck, Darmstadt, Germany). Violaxanthin and phytoene were isolated from natural sources by classical chromatographic techniques [10]. Luteoxanthin, neoxanthin and lutein were obtained as described by Meléndez-Martínez et al. [11].

Trolox (6-hydroxy-2,5,7,8-tetramethylchroman-2-carboxylic acid) was obtained from Fluka Chemika (Neu-Ulm, Switzerland). The reagents 2,2-diphenyl-1-picrylhidracyl radical (DPPH^∙^), monobasic and dibasic sodium phosphate, Folin Ciocalteu’s reagent and fluorescein (free acid) were purchased from Sigma–Aldrich (Steinheim, Germany). Finally, formic acid and solvents (ethanol, methanol, hexane, acetone, diclhoromethane and acetonitrile) were all of analytical grade and were obtained from Merck (Darmstadt, Germany). 

### 2.3. Identification and Quantification of Isoprenoids (Carotenoids and α-Tocopherol) by Rapid Resolution Liquid Chromatography (RRLC)

The extraction and analyses of carotenoids were carried out according to the method described by Stinco et al. [12]. Mango samples (200 mg) were extracted with 1mL of hexane/acetone (1:1 *v*/*v*) using a vortex and an ultrasonic bath for 2 min. Then, samples were centrifuged at 18,000× *g* for 5 min and the colored fractions were recovered. The extraction was performed twice more until color extinction. Finally, the carotenoid extracts were concentrated to dryness in a rotary evaporator at temperature below 30 °C. To obtain saponified carotenoids, the extracts were treated with 1000 µL of dichloromethane and 1000 µL of methanolic KOH (30% *w*/*v*) for 1 h under dim light and at room temperature, after which they were washed with water to remove any trace of base. The extracts obtained were concentrated to dryness in a rotary evaporator and redissolved in ethyl acetate prior to their injection in the RRLC system. Samples were extracted and analyzed in triplicate.

The RRLC acquisitions were made by using an Agilent 1260 system equipped with a diode-array detector, which was set to scan from 200 to 770 nm and a Poroshell 120 C18 column (2.7 µm, 5 cm × 4.6 mm) (Agilent, Palo Alto, CA, USA) kept at 28 °C, according to Stinco et al. [12]. The injection volume was set at 10–20 μL. The mobile phase was pumped at 1 mL/min and consisted of three solvents: solvent A, acetonitrile, solvent B, methanol and solvent C, ethyl acetate. The linear gradient elution was 0 min, 85% A + 15% B; 5 min, 60% A + 20% B + 20% C; 7 min, 60% A + 20% B + 20% C; 9 min, 85% A + 15% B; 12 min, 85% A + 15% B. Chromatograms were monitored at 450 nm. The identification and quantification of isoprenoids were performed by comparison of their chromatographic UV–vis spectroscopic characteristics with the standards, as well as by comparison with the external calibration line calculated. Results were expressed as μg/g dry weight (D.W.).

### 2.4. Folin-Ciocalteu Assay

Folin assay was performed following the method described by Slinkard and Singleton [13]. Briefly, 500 µL of the extracts, blank or standards were placed in a 15 mL tube, where 2.5 mL of the Folin–Ciocalteu reagent was added, allowing to react for 2 min while shaking. Then, 2 mL of a solution of sodium carbonate (75 g/L) was added and properly mixed. The solution was thus incubated 15 min at 50 °C. After that, the absorbance was measured at 750 nm in a spectrophotometer (Shimadzu UV-160A, Kyoto, Japan). Gallic acid was used as a standard (10–90 mg/L) and the results were expressed as mg of gallic acid equivalents (GAE) per gram.

### 2.5. Identification and Quantification of Phenolic Compounds by HPLC–DAD–ESI-MS^n^

Phenolic compounds were extracted and analyzed following the protocol and method of Gironés-Vilaplana et al. [14]. Briefly, samples were extracted with MeOH 70% using the ultrasound technology and kept at 4 °C overnight. Then, samples were filtered and the identification of phenolic compounds was carried out following their MS2 fragmentations by an HPLC–DAD–ESI-MS^n^, constituted by an Agilent series model HPLC (High-performance Liquid Chromatographic) 1100 with a photodiode array detector and a mass spectrometer detector in series (model G2445A) equipped with an electrospray ionization interface (Agilent Technologies, Waldbronn, Germany). The ionization conditions were selected according to those described in the method, covering an *m*/*z* range from 100 to 1200. The acquisition of the mass spectrometry data (MS^n^) was performed in the negative ionization mode for flavonoids, except for anthocyanins, where the positive ionization mode was used. The quantification equipped with a Luna C18 column (25 cm × 0.46 cm, 5 μm particle size) (Phenomenex, Macclesfield, UK) using the acquisition conditions described before. Flavan-3-ols were quantified using the external standard (+)-catechin at 280 nm, flavonols at 360 nm using the standard rutin (quercetin-3-rutinoside) and the anthocyanins by using cyanidin 3-*o*-glucoside at 520 nm. Samples were extracted and analyzed in triplicate. Results were expressed as µg/g D.W.

### 2.6. Antioxidant Capacity

The antioxidant capacity was evaluated using the methods DPPH^∙^ and ORAC, both adapted to a microscale and performed using 96-well micro plates (Nunc, Roskilde, Denmark), which were measured using an Infinite^®^ M200 microplate reader (Tecan, Grödig, Austria). The power of scavenge DPPH radicals were determined according to Mena et al. [15], briefly, 2 μL of the corresponding diluted sample was added to the wells containing 250 μL of DPPH^∙^ dissolved in methanol up to absorbance ~1. Then, the plate was shaken and left for 50 min at 37 °C, thus, the variation in absorbance was measured at 515 nm. Regarding the ORAC method and according to Ou et al. [16], 25 µL of the properly diluted sample was added to 150 μL of fluorescein (1 μM) and, after 30 min of incubation, 25 µL of the radical AAPH (2,2′-azobis(2-methyl-propionamidine)-dihydrochloride) (250 mM) was added to the wells. Results were studied by measuring the variation in fluorescence each 2 min during 120 min of reaction with the radical. Trolox was used as a standard in both methods, following the same procedure as with the samples. Results were expressed as mmol Trolox/100 g D.W.

### 2.7. Statistical Analysis

All assays were conducted in triplicate. The data were processed using the software Statgraphics Centurion version 16.1.18 (Statgraphics.Net, Madrid, Spain). All values were subjected to analysis of variance (ANOVA) with a 95% confidence level. Pearson’s correlation coefficients were also calculated to corroborate relationships among the selected parameters.

## 3. Results and Discussion

The first step to establish the potential applications of the mango by-products, peels and seed kernels, as a valuable source of natural ingredients and/or additives for the pharmaceutical and food industry is crucial to characterize the phytochemical composition with efficient techniques for the identification and quantitation of the nutrients and compounds of interest. Bearing this in mind, different chromatographic methods were used to characterize the isoprenoids, especially carotenoids and α-tocopherol and the phenolic composition of mango by-products.

### 3.1. Isoprenoids: Carotenoids and α-Tocopherol

Many studies have evaluated the carotenoid fraction of mango pulps but not peels and seed kernels. In this sense, this study provides information on the characterization of carotenoid in non-edible parts of the mango. In particular, six carotenoids (4 xanthophylls and 2 carotenes) were identified and quantified in the different mango organs tested (peels, pulps and seed kernels) (Table 1). The carotenoid composition, both qualitative and quantitative, showed differences between the different mango organs tested, which is in line with data previously described in the literature [17]. Violaxanthin and β-carotene stood out as the only two carotenoids present in the different mango fractions tested (Table 1). Regarding pulps and seed kernels, the most abundant carotene was β-carotene, whereas the most important xanthophyll was violaxanthin (Table 1). This is comparable with the results reported by Ornelas-Paz et al. [18] in seven Mexican mango cultivars. Moreover, several authors have described β-carotene as the most predominant carotenoid in Australian and Taiwanese mango pulps [19,20]. In terms of biological effects, β-carotene is considered, theoretically, the carotenoid with the highest provitamin A activity [21] while the health benefits of violaxanthin have yet to be established [17]. With regard to mango peels, lutein highlighted as major carotenoid (Table 1). This agrees with the result reported by Ajila et al. [22] in peels Bandami mango variety. Lutein is an essential nutrient with health promoting effects, especially for eye health. In this line, the use of lutein in the formulation of nutritional supplements have gained increasing popularity for the prevention of age-related macular degeneration, as well as for its antioxidant properties, after the public awareness of its potential to prevent the disease [23]. Thus, the particular interest of the industry, especially pharmaceutical, in the search for new cost-effective sources of lutein as could be mango peels. In addition to its effects on the retina, it has recently been reported the accumulation of lutein in brain, being its content in neural tissue positively correlated with cognitive function, which has intensified interest in identifying functions of lutein in this organ [24]. Furthermore, lutein is a natural colorant, so the mango peels could be employed as additive in the industry such as food, cosmetic and nutraceutical.

On the other hand, the results obtained on the qualitative and quantitative characterization of carotenoid showed differences with the results reported in other mango cultivars (Keitt, Ataulfo, Haden and Kent) from Mexico, Brazil, Taiwan [18,20,25]. These differences could be due to factors such as genetics, agricultural and industrial practices, temperature, harvest, maturity, among others, which can modify the composition of carotenoids [3,17].

The total content of carotenoids (TCC), evaluated as the sum of the content of individual pigments, showed significant differences between the different fractions of mango. The mango pulps showed the highest content (Table 1). TCC obtained in the pulps (14.47 μg/g) is within the range described in other several mango cultivars (9.0 to 92 μg/g) [26]. Although the pulps showed the highest TCC, the result also reflected that mango by-products could be a valuable source of carotenoid, especially mango peels. TCC obtained in mango peels (7.62 μg/g) is higher or comparable than TCC values reported in tropical fruits (Table 2), which reflects that mango peels are not only a disposable waste but an extraordinary source natural of carotenoids. The use of mango peels offers a window of opportunity for configuring alternative food supply chains. The raw material is valuable in terms of nutritional and functional properties and besides, the use of this side stream is of great interest from the point of view of environmental concerns and for food and nutrition purposes.

In relation to α-tocopherol, it is an antioxidant with an effective chemoprotectant agent against lipid oxidation. In the present study, the α-tocopherol was detected and quantified in peels and pulps but not in seed kernels. Concretely, its content was 26 times greater in peels than pulps (Table 1). Abbasi et al. [31] also detected higher content of α-tocopherol in peels than pulps in nine mango cultivars from China. α-tocopherol amount in mango pulps was higher than that described by Burns et al. [32] (0.05 μg/g) in mango from Costa Rica, in agreement with Ornelas-Paz et al. [18] (0.2–0.5 μg/g) in seven Mexican mango cultivars but lower than the content described by Vilela et al. [33] (12–94 μg/g) and Gong et al. [34] (2.0 μg/g) in twelve Portuguese mango cultivars and in mango from China, respectively. As polyphenols and carotenoids, the α-tocopherol amount depends on the genotype, the environmental factors and analytical methods, among other factors [35], which explains the differences observed among the results obtained in this study and others. On the other hand, α-tocopherol concentration obtained in peels was greater than observed in some exotic fruits such as dragon fruit (4.5 μg/g), durian (3.6 μg/g) and papaya (2.6 μg/g) [35]. This result reflects that mango peels could be exploited as natural antioxidant in cosmetic (i.e., anti-aging products), pharmaceutical and agro-industry.

### 3.2. Characterization of the Phenolic Composition

#### 3.2.1. Identification of Phenolic Compounds

**Figure 1:**
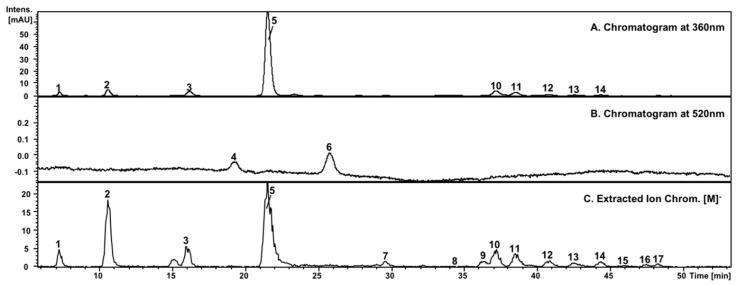
Typical chromatogram of mango organs (e.g., peels), registered at 360 nm (**A**) and 520 nm (**B**) for the identification; Extracted Ion Chromatogram of (M)^−^ of parental ions (**C**) of the phenolic compounds in the mango samples is also included. For the compound assignment numbers, please see Table 3.

Seventeen phenolic compounds were separated and tentatively identified as procyanidins (1–3), anthocyanins (4, 6), xanthones (5, 7, 9, 16, 17) and flavonols (8, 10–15) by HPLC–DAD–ESI/MS^n^, which are shown in Table 3. In addition, the separation of polyphenolics in mango peel is shown in Figure 1.

##### Procyanidins

Peaks 1 and 2 showed the MS spectra of procyanidin dimers with molecular intact ions at *m*/*z* of 423 and 575, respectively and a characteristic deprotonated molecular ion in MS2 of *m*/*z* 289 that corresponds to a (epi)catechin. It is important to highlight that both phenolic compounds had never been determined in mango peels before. On the other hand, peak 3 was identified as a propelargonidin dimer due to its molecular ion (M–H) at *m*/*z* at 559–560 and its MS2/MS3 fragment ions *m*/*z* 407 and 289, corresponding probably to (epi)afzelechin-(epi)catechin. These procyanidins were only characterized in mango peels.

The compounds 4 and 6 exhibited peak absorption maximum at ~280 nm and ~520 nm wavelengths, characteristic of anthocyanins but in extremely low concentration that did not allow the tentative identification of the compounds or the isolation of their aglycones in the (M)^−^ and its corresponding MS/MS fragmentation experiments. These compounds were detected in mango peels but not in pulps and seed kernels.

##### Xanthones

Peak 5 showed a molecular anion at *m*/*z* 421, being therefore tentatively assigned as mangiferin. Peaks 7 and 9, with a corresponding *m*/*z* ion of 573 (M)^−^ and characteristic MS/MS fragmentation, were tentatively identified as mangiferin gallates [36]. In the last part of the chromatogram, tiny peaks of possible isomers of mangiferin (peak 16) and mangiferin gallate (peak 17) were also detected in the MS/MS experiments. These compounds, with exception of mangiferin, were only detected in peels. Mangiferin was also the unique xanthone detected in the pulps of the cultivar Haden, and in the seed kernels of the cultivar Ubá, but the pulps of the cultivar Ubá were constituted by a greater number of xanthones [37]. This result reflects the variability of the phenolic composition in the mango. 

##### Flavonols

Peak 8 was identified as kaempferol derivative according to their UV spectra and MS fragmentation leading to the kaempferol aglycone at *m*/*z* 285 in negative mode [38]. With regard to compounds 10–14, they were identified as quercetin glycosides based on their UV-Vis data and characteristic mass spectra and elution order [39] (Table 3). In the quercetin glycosides, the most abundant fragment ion in MS2/MS3 was *m*/*z* 301 that corresponds with the radical anion of the aglycone quercetin. Peaks 10 and 11 displayed identical (M)^−^ ion, *m*/*z* 463 and could be assigned as quercetin-galactoside and quercetin glucoside, respectively. The formation of the ion at *m*/*z* 433 [M]- as the main fragment ions in the peaks 12, 13 and 14 revealed the presence of three different quercetin pentosides and recognized as quercetin xyloside (12), arabinopyranoside (13) and arabinofuranoside (14), respectively. Finally, the occurrence of an ion (M–H)^−^ at *m*/*z* 447 in the compound 15 indicated the existence of a quercetin rhamnoside. These flavonoids were observed in mango peels but not in pulps and seed kernels. This agrees with previously reported data by Berardini et al. [40] in cultivar Tommy Atkins and Gómez-Caravaca et al. [41] in Keitt mango.

These results reflected that the phenolic profile of peels was different to the profiles obtained in pulps and seed kernels (Table 3). This agrees with information previously reported in the literature, where it is indicated that of different mango phenolics differ in the different plant parts [42]. In particular, in this study the peels showed a higher number of phenolic compounds than pulps and seed kernels. This is in accordance with results previously described in mango [37,43].

#### 3.2.2. Quantification of Phenolic Compounds

The results of the quantification of phenolic compounds in mango are shown in Table 4. Mangiferin was the predominant phenolic compound in the three mango fractions but its quantity was different in each organ. In particular, mango peels presented the highest concentration (2500 μg/g D.W.) followed by seed kernels and pulp (Table 4). This result reflected that non-edible parts of the mango fruit are good sources of mangiferin. Similar results were obtained by Luo et al. [43], Gómez-Caravaca et al. [41] and Ribeiro et al. [37] in 11 Chinese cultivars, cultivar Keitt and cultivar Ubá of mango, respectively. However, the mangiferin concentrations obtained were different to the described by other authors in cultivar Tommy Atkins (peels: 1190.9–1690.4 μg/g; pulps: 2.2 μg/g) [36,43] and cultivars Ataulfo, Keitt, Van Dyke and Ubá, among others (peels: 62.3–21530 μg/g; pulps: not detected-200 μg/g; seed kernels: traces-2340 μg/g) [37,41,43,44,45]. This agrees with data previously reported in the literature, where it has been reported that factors such as cultivar, environment, harvest stage, maturity as well as the method extraction, among other factors, have an effect on the phenolic composition [3,19,44,45]. Regarding the mangiferin biological effects, this phenol possesses an antioxidant capacity higher than other natural antioxidants like vitamin C and E. In this line, this phenolic compound could be used as a food preservative. In addition, mangiferin has a special particular interest for the pharmacological industry by its cancer chemopreventive potential [41].

Regarding the presence of procyanidins, these compounds were found only in the peel and two of them, a catechin derivative (560 μg/g D.W.) and epiafzelechin-epicatechin (600 μg/g D.W.) dimers, constituted the most abundant phenolic compounds after mangiferin (Table 4). These compounds, accounting for the 25% of total phenolic compounds, have been described in mango peels for the first time. According to other authors, these low-molecular weight procyanidins have been described as interesting because of their potent antioxidant capacity and possible protective effects on human health [42,46], especially because dimers can be absorbed intact in the intestinal tract [47] and have recently shown to promote the growth of *Bifidobacterium* in vitro [48]. As natural antioxidants and antimicrobials, proanthocyanidins can be also used in the industry as a preservative, to stabilize food colors, to prevent rancidity due to oxidation of unsaturated fats and to avoid the growth of bacteria and molds [49].

On the other hand, flavonol glycosides obtained in mango peel were identified as quercetin glycosides, being quercetin galactoside and quercetin glucoside the most abundant (Table 4), according to the literature [36,41,50]. Quercetin glycosides biological effects are mainly associated to their antioxidant capacity which could be exploited as a food preservative and stabilizer in the agro-industry, as well as in the development of new drugs in the pharmaceutical industry, among others [51].

The total phenolic content (TPC), evaluated as the sum of the content of individual phenolic, showed significant differences between the different fractions of mango. The order observed was peels > seed kernels > pulps (Table 4). This is in line as described the literature, where it has been reported that phenolic compounds are preferentially located in non-edible parts (peels and seed kernels) and in a lesser extent in the edible part (pulps) [3,42,52].

### 3.3. Antioxidant Capacity and Folin Assay

The health-promoting effects of both bioactive compounds characterized, polyphenols and isoprenoids, have been strongly associated with their antioxidant capacity. Thus, in this present study has been also evaluated the antioxidant capacity of the mango by-products. As shown Table 5, antioxidant capacity of different mango organs (pulp, peel and seed kernel) was evaluated by DPPH radical and ORAC method. Mango by-products highlighted by showing a higher antioxidant capacity than pulps, being seed kernels the mango fraction that exhibited the highest values of antioxidant capacity. The order described is comparable with that obtained by Abbasi et al. [30], Guo et al. [53] and Sogi et al. [54] in nine Chinese mango cultivars, in Chinese mango cultivar and in Tommy Atkins mango from USA, respectively. However, the values obtained in mango peels and seed kernels were lower than that described by Sogi et al. [54] (seed kernels = 154.7–181.9 mmol Trolox/100 g; peels = 41.8–77.6 mmol Trolox/100 g), while mango pulp was higher than that reported by Noratto et al. [55] in five Mexican mango cultivars (15.0–32.7 mmol Trolox/100 g). In this line, it has been described that mango antioxidant capacity can be affected by different factors such as variety, maturity, agricultural and industrial practices [36]. Moreover, it is important to indicate that ORAC value of seed kernels was greater than the ORAC values reported in other tropical fruits with demonstrated high polyphenol content such as banana passion fruit and Andean blackberry [56,57]. These results suggested that mango by-products, especially seed kernels, could represent a valuable ingredient with high antioxidant capacity in the agro-industry. In spite of this, it is important to note that the results were obtained by in vitro methods, which do not take into account the (among others) the metabolic transformations and interactions that clearly affect the bioavailability and biological action of phytochemicals [58,59]. In particular, the predominant forms of polyphenols in plasma are conjugates (glucuronates or sulfates, with or without methylation), which are chemically distinct from their parent compounds and therefore their properties are also different [59]. Thus, prior to the use of mango by-product as a new ingredient in the industry is necessary to go deeper into the analysis of their antioxidant activity and carry out assays of antioxidant capacity in vivo that corroborate the results obtained in vitro; as well as analyze the synergism or antagonism between the bioactive compounds present in the mango by-product matrix both at the level of bioavailability and biological action.

On the other hand, the order observed in Folin-Ciocalteu assay by mango organs, seed kernels (23,759.13 mg/100 g GAE) > peels (2,874.97 mg/100 g GAE) > pulps (563.01 mg/100 g GAE), coincided with that described in the antioxidant activity. This result makes sense, because Folin-Ciocalteu assay determines the content of polyphenols and other reducing bioactive compounds (sugars, amino acids, etc.) that have antioxidant properties.

Finally, the correlation phytochemical composition (polyphenol, carotenoid and α-tocopherol) versus antioxidant capacity (DPPH and ORAC) was positive and direct (Table 6).

## 4. Conclusions

In summary, the results obtained demonstrate that the non-edible parts of mango, the peels and seed kernels commonly managed as a waste in the industry, are a good source of bioactive compounds like isoprenoids, especially carotenoids and α-tocopherol and polyphenols with high nutritional value and health benefit effects. In particular, mango peels constituted the great part of the fruit in terms of bioactive compounds contents, following by the seed kernels and both compared to the pulp, being some of these components essential nutrients in the human diet, such as lutein and α-tocopherol. In addition, mango by-products, especially seed kernels, exhibited higher antioxidant activity than pulps. Therefore, mango by-products could be exploited as a natural preservative by the pharmaceutical and agro-food industry, as well as being used as a natural ingredient contributing to different health benefits to consumers. Besides, the amount of waste generated during the processing of the mango could be reduced. Finally, future studies will be carried out deeply to elucidate the bioavailability and safety of bioactive compounds of mango by-products.

## Figures and Tables

**Table 1:** Isoprenoid, carotenoids and α-tocopherol, composition in mango organs (peels, pulps and seed kernels).

	Concentration (μg/g D.W.)		
	Peel	Pulp	Seed Kernel
*Carotenoids*			
Violaxanthin *	1.58 ^b^ ± 0.13	3.97 ^a^ ± 0.19	0.18 ^c^ ± 0.02
Lutein	3.26 ± 0.19	-	-
Luteoxanthin	-	1.69 ^a^ ± 0.08	0.16 ^b^ ± 0.04
β-cryptoxanthin	-	2.72 ± 0.04	-
β-carotene	2.78 ^b^ ± 0.05	4.86 ^a^ ± 0.01	0.50 ^c^ ± 0.01
Phytoene	-	1.23 ^a^ ± 0.01	0.23 ^b^ ± 0.03
*∑carotenoids*	7.62 ^b^ ± 0.37	14.47 ^a^ ± 0.33	1.07 ^c^ ± 0.10
α-tocopherol	10.20 ^a^ ± 1.13	0.39 ^b^ ± 0.21	-

* In peel, the concentration of violoxanthin corresponds to violaxanthin + neoxanthin; ^a–c^ Mean values with different letter on the right in the same row indicate statistically significant differences among the three treatments (*p* < 0.05).

**Table 2:** Carotenoid concentration in tropical fruits.

	Carotenoid Concentration (μg/g D.W.)
*Eugenia stipitata*	8.06 [27]
*Solanum quitoense*	7.94 [28]
*Ananas comosus*	4.97 [29]
*Psidium guajava*	6.04 [29]
*Carica papaya*	7.93−51.34 [30]

**Table 3:** Tentative identification of phenolic compounds in mango organs (peels, pulps and seed kernels) by HPLC–DAD–ESI-MS^n^.

Peak Number	Rt (min)	DAD (Max. Abs.) λnm	(M)^−^	Fragment Ions (MS^n^)	Phenolic Compounds (Tentative Identification)	Peels	Pulp	Seed Kernels
1	7.2	280	423	303, 289	Procyanidin (catechin derivative)	√	-	-
2	10.5	280	575	423, 289	Procyanidin dimer	√		
3	15.9	280	559	407,289	(Epi)afzelechin-(epi)catechin dimer	√	-	-
4	19.2	280, 520	-	-	Unidentified anthocyanin	√	-	-
5	21.5	330,360	421	403, 331, 301, 258–259	Mangiferin	√	√	√
6	25.7	280, 520	-	-	Unidentified anthocyanin	√	-	-
7	29.5	360	573	421, 403, 331, 301	Mangiferin gallate	√	-	-
8	34.4	360	599	285	Kaempferol derivative	√	-	-
9	36.3	360	573	421, 403, 331, 301	Mangiferin gallate	√	-	-
10	37.1	360	463	301	Quercetin galactoside	√	-	-
11	38.5	360	463	301	Quercetin glucoside	√	-	-
12	40.8	360	433	301	Quercetin xyloside	√	-	-
13	42.5	360	433	301	Quercetin arabinopyranoside	√	-	-
14	44.4	360	433	301	Quercetin arabinofuranoside	√	-	-
15	45.8	360	447	301	Quercetin rhamnoside	√	-	-
16	47.4	330,360	421	403, 373, 331, 301	Mangiferin (isomer)	√	-	-
17	48.2	360	573	421, 403, 331, 301	Mangiferin gallate (isomer)	√	-	-

Rt: retention time; DAD: dyode-array detrector.

**Table 4:** Concentration of phenolic composition in mango peel, pulp and seed kernel.

*Peak*	*Phenolic Compounds*	Concentration (μg/g D.W.)		
		Peel	Pulp	Seed Kernel
1	Procyanidin (catechin derivative)	560 ± 60	-	-
2	Procyanidin dimer	<LOQ	-	-
3	Epiafzelechin-epicatechin dimer	600 ± 60	-	-
4	Unidentified anthocyanin	<LOQ	-	-
5	Mangiferin	2500 ^a^ ± 320	50 ^c^ ± 20	430 ^b^ ± 90
6	Unidentified anthocyanin	30 ± 0	-	-
7	Mangiferin gallate	<LOQ	-	-
8	Kaempferol derivative	<LOQ	-	-
9	Mangiferin gallate	<LOQ	-	-
10	Quercetin-galactoside	220 ± 20	-	-
11	Quercetin glucoside	180 ± 10	-	-
12	Quercetin xyloside	<LOQ	-	-
13	Quercetin arabinopyranoside	80 ± 10	-	-
14	Quercetin arabinofuranoside	50 ± 0	-	-
15	Quercetin rhamnoside	50 ± 0	-	-
16	Mangiferin (isomer)	<LOQ	-	-
17	Mangiferin gallate (isomer)	<LOQ	-	-
	*∑phenolic compounds*	4270 ^a^ ± 480	50 ^c^ ± 20	430 ^b^ ± 90

LOQ: limit of quantification; ^a–c^ Mean values with different letter on the right in the same row indicate statistically significant differences among the three treatments (*p* < 0.05).

**Table 5:** Antioxidant capacity in mango organs (peels, pulps and seed kernels).

	Antioxidant Capacity (mmol Trolox/100 g D.W.)		
	Peel	Pulp	Seed Kernel
DPPH	11.06 ^b^ ± 0.42	2.11 ^c^ ± 0.12	44.34 ^a^ ± 0.89
ORAC	29.87 ^b^ ± 2.69	1.83 ^c^ ± 0.45	126.08 ^a^ ± 2.44

^a–c^ Mean values with different letter on the right in the same row indicate statistically significant differences among the three treatments (*p* < 0.05).

**Table 6:** Pearson’s correlation coefficients (*r*) between bioactive compounds (isoprenoids polyphenols and) in mango organs (peels, pulps and seed kernels) and its antioxidant capacity (DPPH and ORAC).

	Bioactive Compounds Mango		
Assay	Peel	Pulp	Seed Kernel
DPPH	0.97	0.89	0.92
ORAC	1.00	0.90	0.97
